# Risk Factors, Distribution Patterns, and Their Implications for Environmentally Based Tuberculosis Transmission Risk Control Strategies

**DOI:** 10.3390/ijerph23081063

**Published:** 2026-08-17

**Authors:** Rapitos Sidiq, Indang Dewata, Heldi, Nurhasan Syah, Linda Handayuni, Al Asyary

**Affiliations:** 1 Padang Health Polytechnic, Indonesian Ministry of Health, Padang 25146, Indonesia; 2 Faculty of Mathematics and Natural Sciences, Universitas Negeri Padang, Padang 25131, Indonesia; indangdewata@fmipa.unp.ac.id; 3 Faculty of Language and Art, Universitas Negeri Padang, Padang 25131, Indonesia; enstenheldi@fbs.unp.ac.id; 4 Faculty of Civil Engineering, Universitas Negeri Padang, Padang 25131, Indonesia; nurhasan@ft.unp.ac.id; 5 College of Health Sciences, Dharma Landbouw, Padang 25133, Indonesia; lindahandayuni@gmail.com; 6 Department of Environmental Health, Faculty of Public Health, Universitas Indonesia, Depok 16424, Indonesia

**Keywords:** tuberculosis, environmental epidemiology, spatial analysis, multidimensional scaling model, sustainability, community triggering, public health

## Abstract

**Highlights:**

**Public health relevance—How does this work relate to a public health issue?**

- Tuberculosis remains a major public health problem in high-burden areas, where environmental conditions play a role in maintaining transmission.
- Identification of environmental risk factors and case distribution patterns is important to strengthen evidence-based TB transmission control strategies.

**Public health significance—Why is this work of significance to public health?**

- This study measures the magnitude of the association between modifiable environmental risk factors and TB incidence by controlling for confounding variables.
- Analysis of distribution patterns provides epidemiological evidence to support more targeted environmentally based TB risk control interventions.

**Public health implications—What are the key implications or messages for practitioners, policy makers, and/or researchers in public health?**

- TB control programs need to integrate environmental risk mitigation into primary health care and national TB strategies.
- Environmentally based interventions and community empowerment have the potential to increase the sustainability of TB transmission prevention in high-risk areas.

**Abstract:**

Tuberculosis (TB) remains one of the most burdensome infectious diseases in Indonesia. One contributing factor is the environment, particularly issues related to household ventilation and lighting. TB control efforts do not solely rely on medical interventions but also require a community-based approach that emphasizes active community involvement in preventing and controlling environmental risks to TB transmission. This study aims to analyze risk factors, distribution patterns, and their implications for environmentally based TB transmission risk control strategies. A mixed-methods design was conducted in two stages: first, risk factor analysis (with a case–control approach) and disease distribution pattern determination on environmental variables were performed, followed by a multidimensional scaling model (by using the Rapid Appraisal for Fisheries (RAPFISH) method) according to sustainability dimensions (ecological, economic, social, institutional, and technological) based on the resulting significant variables/indicators. This research was conducted in 2024, in four villages/nagari in West Sumatra of Indonesia. For quantitative analysis, data were collected through interviews, physical measurements of the environment, and determination of the coordinates of the residences of people with TB. Data were analyzed using bivariate and multivariate analyses. For the distribution pattern, Nearest Neighbor Analysis (NNA) in the ArcView program version 3.1 was used. Meanwhile, for qualitative data collection, focus group discussion (FGD) was performed. This study found that poor ventilation, minimal lighting, and a lack of stigma competency significantly increased the risk of TB transmission. Varied distribution patterns between regions demand adaptive and locally based strategies. The community-triggering approach is key to building community awareness and participation in creating a healthy and TB-free home environment.

## 1. Introduction

Tuberculosis (TB) remains one of the significant health problems in the world, especially in Indonesia. Data from the Ministry of Health in 2023 showed that Indonesia had the second-highest TB caseload in the world [1,2,3]. This disease impacts not only individual health, but also the social and economic aspects of society [4]. Environmental elements, such as biotic, abiotic, and cultural elements, greatly influence the high rate of TB transmission. Abiotic factors, or the physical environment, such as inadequate housing conditions, high population density [5,6], and cramped homes, increase the risk of TB transmission, especially in children [7,8,9]. Additionally, homes characterized by poor temperature regulation, humidity control, lighting quality [10,11], and poor air circulation [5,12], as well as exposure to air pollution [13] and cigarette smoke [14], further exacerbate the issue [15,16]. Therefore, the physical condition of a home serves as an important indicator of TB transmission risk [17].

One of the biggest challenges in controlling TB is the low public awareness regarding environment-based prevention efforts [18]. The Indonesian government, through the 2020–2024 National TB Elimination Strategy, has emphasized the importance of community involvement in controlling TB disease [19]. Consequently, it is necessary to conduct a study of the risk factors for transmission and the pattern of TB case distribution. This study is vital in determining the form of environmental control strategies and changes in community behavior. The study was conducted in four villages, or nagari (village), which reported the highest TB cases in absolute numbers, located in both highlands and coastal areas of four regencies/cities in West Sumatra. Its objective was to analyze the risk factors of TB transmission, distribution patterns, and their implications for environmentally based TB transmission risk control strategies.

## 2. Materials and Methods

### 2.1. Study Design and Setting

This study used a mixed-methods approach with a quantitative design and a case–control approach, complemented by qualitative elements through the multidimensional scaling (MDS) model, using the RAPFISH method. For quantitative analysis, the sample size was calculated using the minimum sample size of WHO-Lemeshow, with a 5% alpha and 80% power of tests for a case–control study with a 1:2 comparison, according to previous point and parameter estimations from the related region in Indonesia [20]. 

It consisted of 36 people with TB, while the controls were healthy individuals living in the same environment as the patients, totaling 72 people, with a ratio of 1:2 for the number of cases to controls. Controls were individuals located in the same household who had negative results for latent TB infection during the regular TB contact investigation when the TB case was confirmed. This study involved participants with TB in the third trimester of 2024, and all study phases were conducted from January to October, 2024, in four villages: (1) Nan Balimo Village, Tanjung Harapan District, Solok City; (2) Biaro Gadang Village, Ampek Angkek District, Agam Regency; (3) Surantih Village, Sutera District, Pesisir Selatan Regency; and (4) Gates Nan XX Village, Lubuk Begalung District, Padang City—West Sumatra of Indonesia.

### 2.2. Data Collection and Analysis

Data were collected through interviews, physical measurements of the environment, and the determination of the coordinates of the residences of people with TB. Validated disease psychosocial environment information related to people with TB, defined as TB stigma competency (Appendix A), was adopted from the national guideline for TB prevention [19]. Both relative humidity and home temperature were measured using a thermohygrometer, while lighting was measured using a lux meter and ventilation with a tape measure. All tools were certified by the Directorate of Metrology, Indonesian Ministry of Industry. Also, all calculations of these data are averages of 3 measurements taken during the daytime. Data were analyzed using bivariate and multivariate methods (Chi-square and logistic regression).

For the pattern of case distribution, data analysis was carried out using the ArcView program version 3.1, which is a tool for creating, analyzing, and visualizing spatial and geographic data. Nearest Neighbor Analysis (NNA) was used with the following formula [21]:

*R*n = 2D √(*n*/*a*)

where Rn is the nearest neighbor index, D is the average distance between each point and its nearest neighbor, n is the number of points studied, and a is the area. NNA produces an Rn (nearest neighbor index) value that measures whether the pattern of case transmission is clustered, random, or regular. A clustered pattern is indicated by an Rn value of 0, meaning all points are adjacent to one point. Meanwhile, a random pattern shows an Rn value of 1.0, indicating there is no clear pattern. A regular pattern is indicated by an Rn value of 2.15, meaning a perfectly uniform distribution where each point is equidistant from its neighbors. The main data entered in the analysis process are related to geographic location, such as latitude and longitude coordinates, and point size. The data are presented in the form of a frequency distribution table and a map.

The multidimensional scaling (MDS) model uses the scale to measure the distance between points [22]. The RAPFISH provides this principle as the statistical computation to determine the sustainability of fisheries, developed by the University of British Columbia Fisheries Center [23]. In its development, the RAPFISH is applied not only to the fisheries sector but also to all sustainable development sectors [24]. In this study, this tool is developed to determine the sustainability of environmentally based TB transmission risk control strategies.

In this study, the RAPFISH method consists of five dimensions: ecological, economic, social, institutional, and technological. Expert assessments were conducted using purposive sampling, involving experts in environmental science, public health, sociology, government, and environmental health, including physicians (Appendix B). The methods used were focus group discussions (FGDs) and questionnaires.

### 2.3. Ethics Approval and Consent to Participate

This study obtained research ethics approval from the Ethics Committee of Health Research at the Indonesian Public Health Association, with an ethics approval number of 124/KEPK-IAKMI/VI/2020.

## 3. Results

### 3.1. Risk Factors for TB Transmission

Based on Table 1, stigma competency was observed to be mostly in the high category (58.25%). The highest levels were found in the Gates Nan XX and Nagari Surantih subdistricts, each at 30%. For the abiotic or physical environmental factor of room humidity, 82.4% was between the 40% and 60% category, with the highest level in the Nan Balimo subdistrict at 30.3%. In terms of temperature, the majority (61.1%) were in the category of less than 18 °C and more than 30 °C, with the highest recorded in the Biaro subdistrict. Regarding home lighting, most measurements were in the category of more than or equal to 60 Lux (63.9%), with the highest being in the Nan Balimo subdistrict at 27.5%. Finally, for the ventilation size, most were categorized as being more than 10% of the floor area, with the highest in the Nan Balimo subdistrict at 31%.

According to Table 2, three variables exhibited a significance value of less than 0.05 and an Odds Ratio (OR) exceeding 1. These variables were ventilation size (OR = 8.322), stigma (OR = 9.074), and lighting (OR = 9.013).

### 3.2. TB Spread Pattern

**Figure 1:**
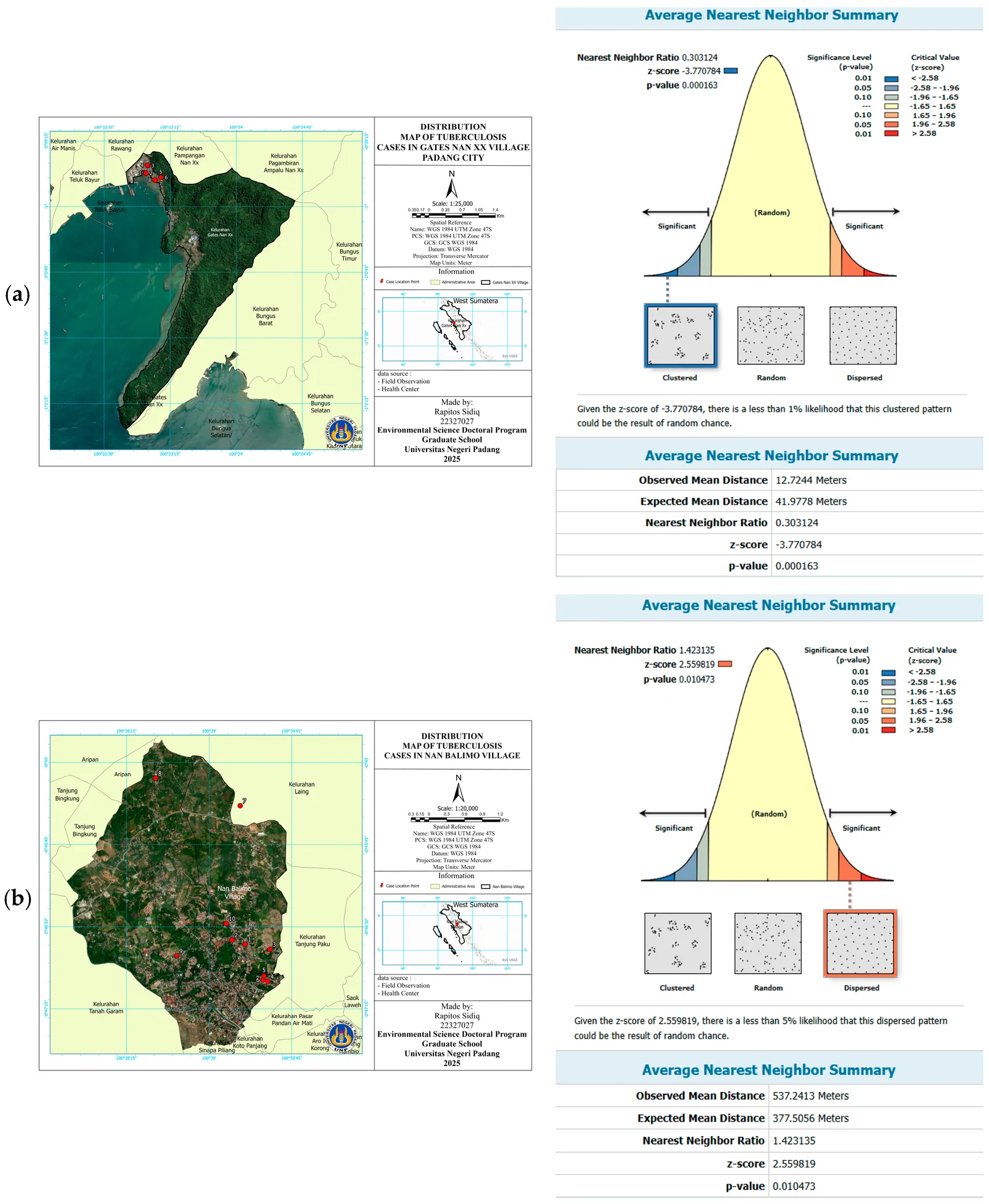

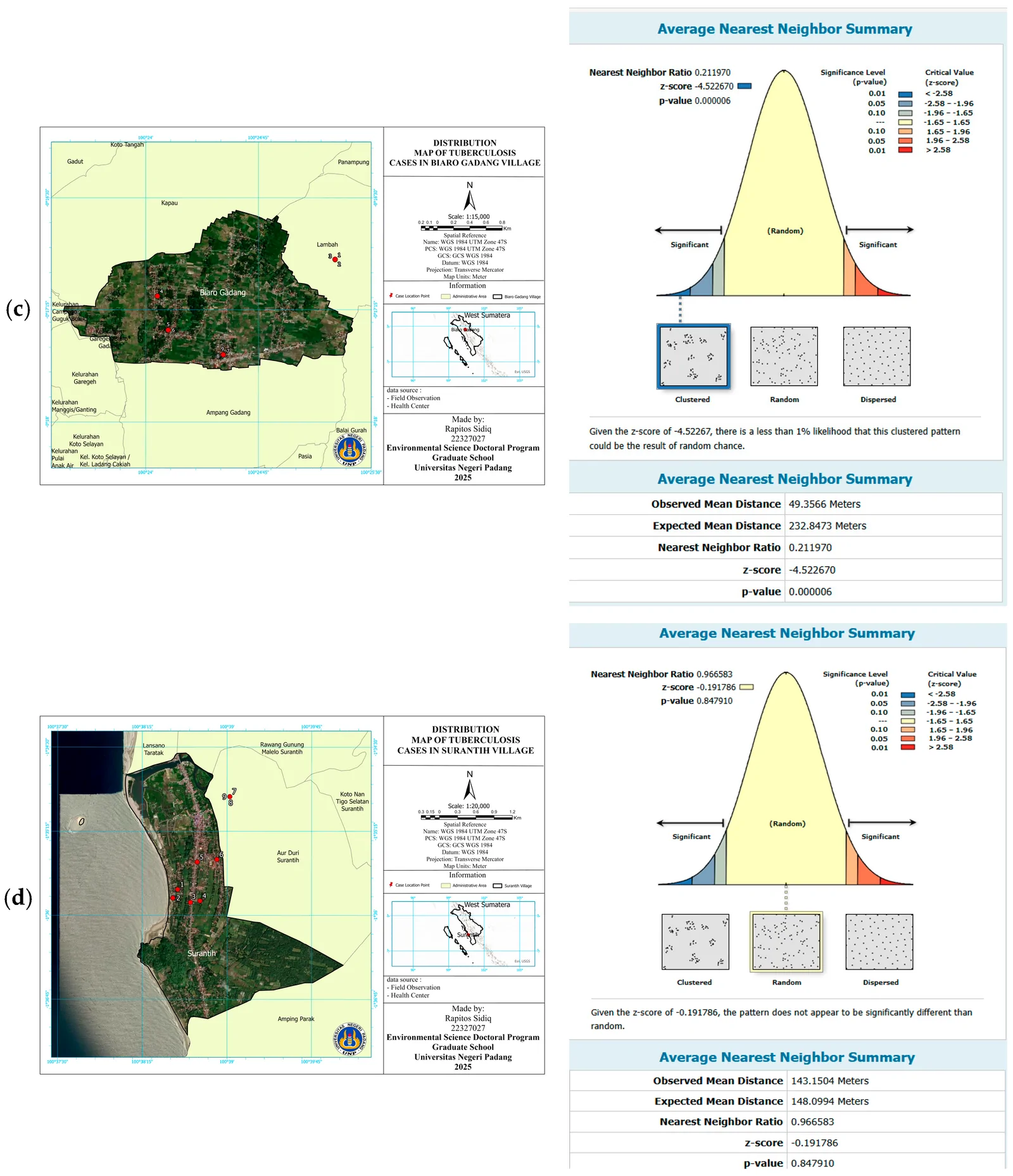
Distribution pattern of pulmonary TB cases in: (**a**) Gates Nan XX of Padang City, (**b**) Nan Balimo subdistrict of Solok City, (**c**) Biaro Gadang Village of Agam Regency, and (**d**) Surantih Village of Pesisir Selatan Regency—Indonesia.

Based on Figure 1a, there were eight cases of pulmonary TB in this subdistrict. Meanwhile, the NNA showed that the pattern of distribution of pulmonary TB cases in Gates Nan XX Village was clustered or in groups.

**Figure 2:**
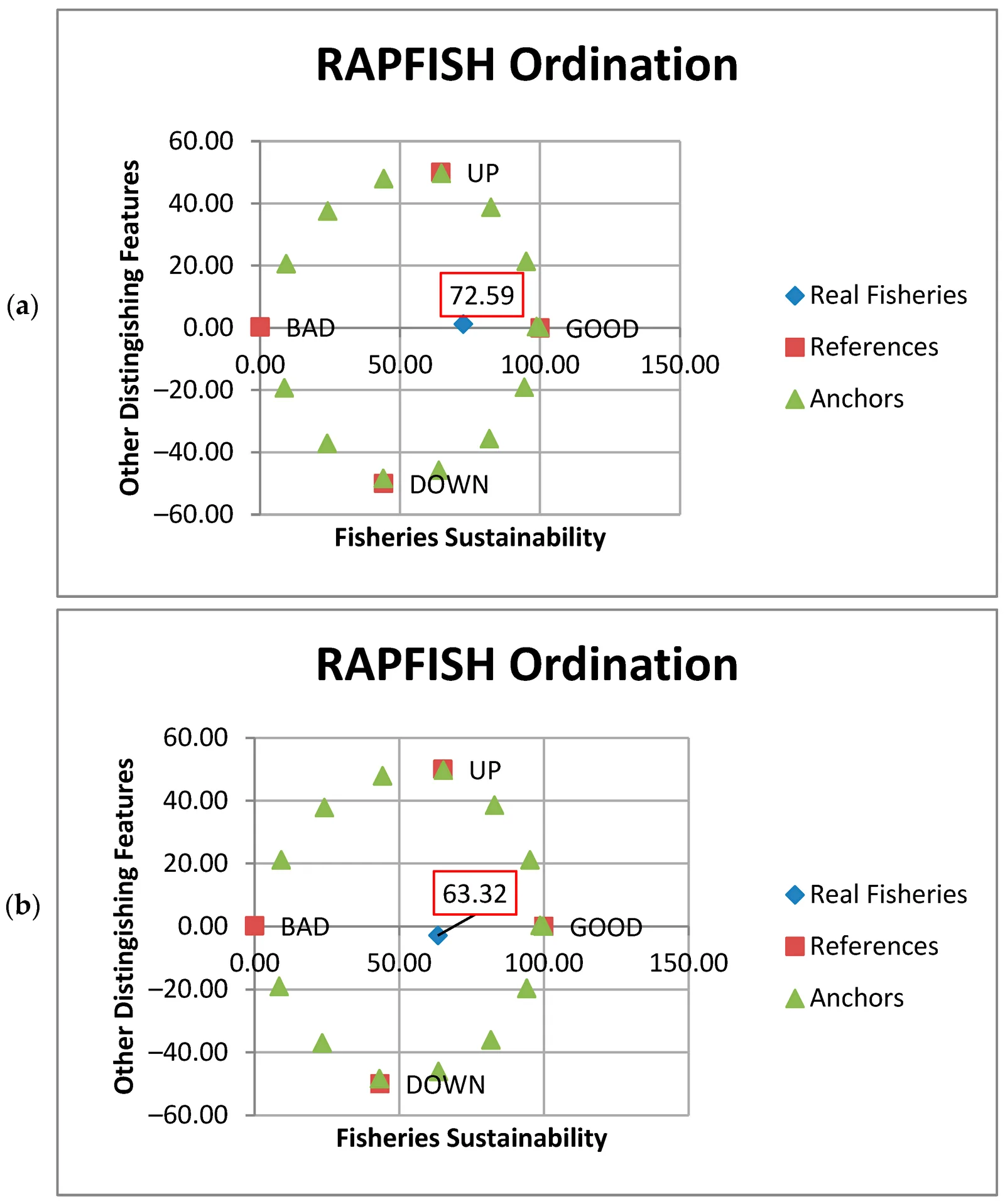

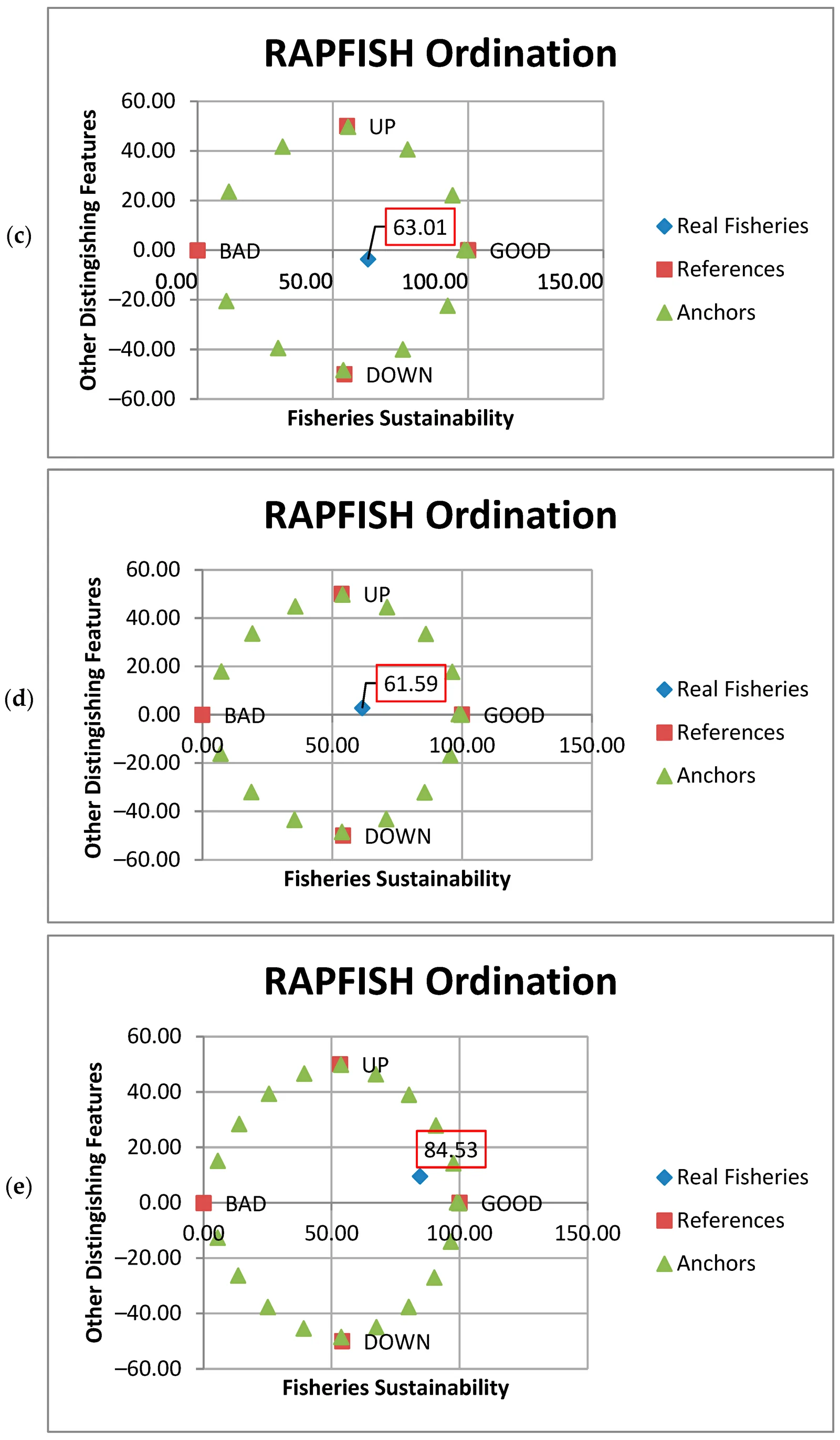
Sustainability status of community triggers from (**a**) ecological, (**b**) economic, (**c**) social, (**d**) institutional, and (**e**) technological dimensions.

Based on Figure 1b, there were 10 cases of pulmonary TB in the Nan Balimo subdistrict. According to NNA, the distribution pattern of pulmonary TB cases in Nan Balimo Village was dispersed. This dispersed pattern indicates that pulmonary TB cases were spread out and far from each other, rather than being concentrated in one location or area. Figure 2c shows that nine pulmonary TB cases were identified in the Biaro Gadang Village. Based on NNA results, the distribution pattern of pulmonary TB cases in this village was clustered or grouped, with cases concentrated in certain areas. Based on Figure 2d, nine pulmonary TB cases were found in Nagari Surantih. Based on NNA, the distribution pattern of pulmonary TB cases in this nagari was random or haphazard, namely with an unclear pattern.

The analysis using the RAPFISH method, whose validity was confirmed through Monte Carlo sensitivity testing, determined the sustainability index values for the ecological, economic, social, institutional, and technological dimensions to be 72.59, 63.32, 63.10, 61.59, and 85.53, respectively (Figure 2).

In Figure 2, the score was obtained within the context of an environment-based pulmonary TB transmission risk control strategy, specifically through a community-triggering approach related to home ventilation, lighting, and stigma (Figure S1). Based on the classification range (51–75), this score categorizes the ecological sustainability status at the “moderately sustainable” level. It is aligned with the economic, social, and institutional dimensions. Meanwhile, the technological dimension’s classification range (76–100) indicates that the technology sustainability status is at the “highly sustainable” level.

A summary of the results of the multidimensional sustainability analysis can be seen in Table 3 as follows:

Based on Table 3, the average sustainability index for the pulmonary TB transmission risk control strategy was 69.01. This value places the overall program sustainability status in the “Moderately Sustainable” category, with a score range of 51–75. The quality of the model used was confirmed by stress values, all of which were lower than the threshold of 0.25, and a coefficient of determination (R^2^) value approaching 1. These two indicators suggest that the analysis model exhibits excellent stability and goodness of fit, indicating that the analyzed attributes are suitable. Furthermore, a comparison between the MDS ordination results and the Monte Carlo simulation at the 95% confidence level revealed a relatively small difference in the values. This insignificant difference indicates that the potential for error in attribute weighting and the overall analysis procedure is minimal. Therefore, this model is considered highly representative and reliable in capturing the sustainability of the pulmonary TB transmission risk control strategy. The analysis results indicate that of the five dimensions, four—ecology (72.59), economy (63.32), social (63.01), and institutional (61.59)—are in the “Moderately Sustainable” category, while the technological dimension shows the best performance with a value of 84.53 and is classified as “Highly Sustainable,” making it the most dominant dimension in this strategy. In the technical aspect of modeling, the stress value for each dimension ranges from 0.134 to 0.160, all of which are below the tolerance limit of 0.25. In line with this, the R^2^ value shows excellent results, ranging from 0.939 to 0.953. This indicates that the multidimensional scaling (MDS) model used has high accuracy and reliability, and thus, the attribute set used is adequate. The Monte Carlo simulation results further strengthen the reliability of the analysis by showing a relatively small difference in values compared to the MDS ordination results. This minimal difference reflects that the error rate in the evaluation process is very low; hence, the model used can be considered valid, stable, and representative in describing the actual conditions of the sustainability of the TB transmission risk control strategy (Figure S2).

**Figure 3:**
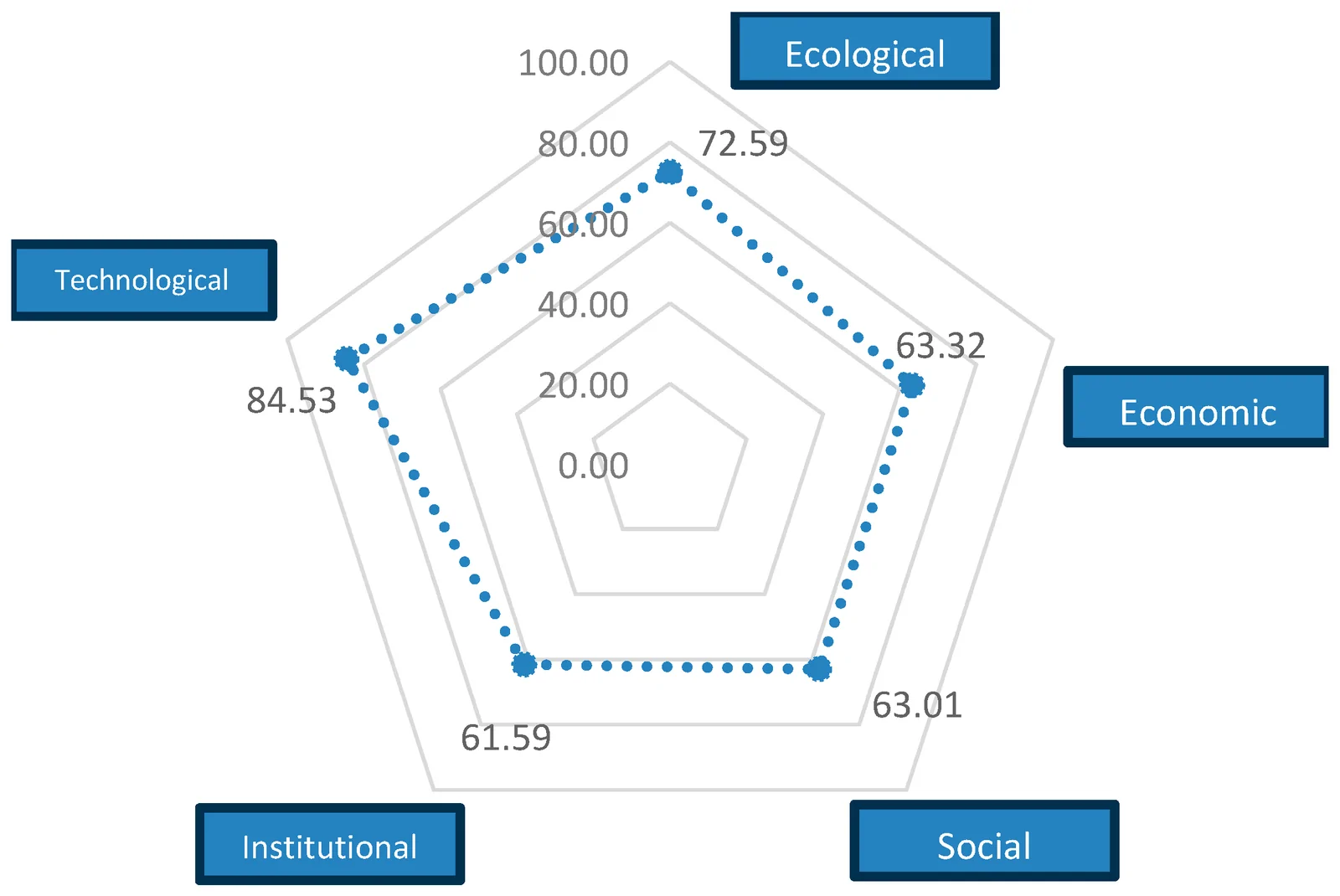
Diagram of the sustainability status of community triggering as an environment-based pulmonary TB transmission risk control strategy.

Figure 3 illustrates that the sustainability status values from the RAPFISH analysis for each dimension are mapped in a diagram to provide a more comprehensive visualization of the overall sustainability status of the pulmonary TB transmission risk control strategy. This visualization encompasses the various dimensions analyzed: ecological, economic, social, institutional, and technological.

## 4. Discussion

The results of this study emphasize that home environmental factors and social aspects are significantly associated with the risk of TB transmission. The findings indicated that the home ventilation size (OR = 8.322), natural lighting (OR = 9.013), and stigma competency of people with TB (OR = 9.074) were significantly associated with the risk of TB transmission. This emphasizes that TB control must be designed holistically, going beyond medical interventions, and encompassing the physical aspects of the disease and the social dynamics of the community. Poor ventilation has a direct impact on the concentration of droplet nuclei containing *Mycobacterium tuberculosis* in a confined space. Previous studies have shown that inadequate ventilation with openings less than 10% of the floor area is associated with a 7–10-fold increased risk of TB [25]. The World Health Organization (WHO) has emphasized in its technical guidelines that adequate natural ventilation is a key element in healthy home design in densely populated areas [26]. Natural lighting has also been shown to act as a protective factor, through exposure to ultraviolet light that can reduce the viability of pathogen in the air and on surfaces [27]. A study by Alamzarwati et al. (2026) found that homes with adequate lighting have a 72% reduced risk of TB [28]. This highlights the importance of adopting a tropical architectural style approach that emphasizes natural lighting as part of biological mitigation against infection.

In addition, stigma has emerged as a major obstacle to TB prevention and treatment [4]. The high OR shows that individuals living in stigmatized environments are more likely not to report symptoms, hesitate to seek treatment, and resist making changes to create a healthy home environment. The WHO also highlights that stigma remains a major challenge in TB control efforts, especially in developing countries [18]. From a spatial perspective, the variations in TB case distribution patterns identified in the four study areas (two clustered, one dispersed, and one random) have provided direct implications for neighborhood-based intervention strategies. The clustered patterns found in coastal and highland areas indicated a concentration of cases in densely populated areas, likely driven by intense social interaction, poor ventilation, and limited access to information [29]. In this context, control strategies should be targeted, such as implementing intensive community interventions in priority zones, renovating collective ventilation systems, and establishing neighborhood-based health education centers. For dispersed areas, where cases were widespread, control approaches need to emphasize personalized interventions that directly target families. Interventions such as home visits by neighborhood cadres, healthy home monitoring, and personal narrative-based education are more effective in increasing awareness and behavioral change [30]. Meanwhile, the random patterns indicate the potential for transmission driven by high social mobility and the absence of centralized transmission sources. Control strategies in this region should incorporate adaptive spatial monitoring systems and encourage the implementation of healthy home audits that align with the village policies. The WHO recommends implementing a spatial early detection system in unstructured conditions to prevent a surge in cases [31]. Other than technical interventions, a community-based participatory approach is essential for increasing public awareness. Community-triggering activities based on local risk mapping have been shown to increase community ownership and involvement in improving the home environment [32]. The involvement of local leaders, youth, and social institutions in creating joint solutions has been proven to strengthen social resilience and support a community-oriented health system.

Therefore, considering both the physical aspects of the home, social factors, and spatial distribution patterns, an environmentally focused TB control approach must be adapted to the local context. Strategies that account for the characteristics of case distribution, household conditions, and community awareness can promote long-term success in TB transmission prevention efforts. One such environmentally based strategy for controlling the risk of TB transmission is community sensitization. Community triggering is a participatory approach that can raise critical awareness, foster a sense of ownership of the problem at the community level, and encourage awareness-based collective action. This strategy aims to strengthen conventional public health promotion activities (e.g., through counseling, leaflets, posters), which are often one-way and informative, but do not necessarily inspire behavioral change. Therefore, this approach is environment-based, focusing on identified risk factors such as ventilation and lighting conditions. It supports local capacity building and social adaptation, thereby strengthening community resilience to pulmonary TB [33,34].

The study indicates that the contribution of environmental aspects to this strategy still requires further optimization through strategic interventions and by strengthening community and institutional capacities for ecological management.

Similarly, the economic aspect needs additional reinforcement through targeted and strategic interventions, enhancement of community economic capacity, and sustained financial support from relevant institutions.

From a social standpoint, the strategy would benefit from stronger community engagement and the cultivation of social capital through institutional partnerships and programs that encourage active participation.

Institutionally, improvements should focus on strengthening regulations, enhancing coordination among institutions, and building the capacity of relevant institutions to ensure long-term sustainability.

In contrast, the contribution of technology is already substantial. The primary focus, moving forward, should be to maintain this level of achievement through regular technology updates and ensuring their accessibility for public use. To further enhance technological sustainability, strategic priorities should emphasize strengthening digital data systems, providing institutional support for challenging areas, and ensuring the availability of resources and equipment.

Two attributes emerged as the most influential levers for improving ecological sustainability. Consequently, intervention strategies should be tailored to the specific conditions of the location and focus on efforts to maintain consistent healthy behavior changes in the community.

Policies should prioritize improving the sustainability status of the economic dimension, and special attention needs to be paid to ensuring the cost efficiency of the program and maximizing its long-term economic benefits.

These findings suggest that to enhance the social sustainability of programs effectively, intervention strategies must prioritize the involvement and strengthening of local leaders as key drivers.

On the other hand, these findings also suggest that the most effective strategy to ensure the program’s sustainability from an institutional perspective is to integrate it deeply into the social structures and activities that are already operating routinely in the community.

The root causes of this environmentally based strategy for mitigating the risk of pulmonary TB transmission are identified as stemming from several critical factors across each dimension of sustainability. These sensitive factors, encompassing the ecological, economic, social, institutional, and technological dimensions, must be a top priority in formulating effective interventions. Therefore, policies focused on these key elements are essential to improve the program’s sustainability comprehensively. Successful pulmonary TB control relies heavily on multi-stakeholder collaboration, including the community, government, private sector, and the use of appropriate technology. Therefore, a holistic and integrated approach is essential for designing effective strategic steps. This integrated approach is crucial for anticipating cascading effects, where weaknesses in one dimension can trigger declining performance in others. Without comprehensive, cross-sectoral action, there is a significant risk of diminishing the overall sustainability of the pulmonary TB control program.

## 5. Conclusions

Poor ventilation, minimal lighting, and psychosocial stigma competency have been shown to be associated with the risk of TB transmission significantly. Varied distribution patterns between regions demand adaptive and locally based strategies. The community-triggering activities strategy is essential for fostering community awareness and encouraging participation to create a healthy, TB-free home environment.

## Figures and Tables

**Table 1:** Frequency distribution of environmental factors based on research location.

Environmental Aspects	Category	Locations								Amount	%
		Biaro		Gates Nan XX		Nan Balimo		Surantih			
		n	%	n	%	n	%	n	%		
Stigma Competency	High	10	16.7	18	30.0	14	23.3	18	30.0	60	58.25
	Low	16	37.2	5	11.6	15	34.9	7	16.3	43	41.75
Room Humidity	Less than 40% and more than 60%	8	42.1	3	15.8	3	15.8	5	26.3	19	17.6
	Between 40% and 60%	19	21.3	21	23.6	27	30.3	22	24.7	89	82.4
Home Temperature	Less than 18 °C and more than 30 °C	20	30.3	18	27.3	12	18.2	16	24.2	66	61.1
	Between 18 °C and 30 °C	7	16.7	6	14.3	18	42.9	11	26.2	42	38.9
Home Lighting	Less than 60 Lux	11	28.2	8	20.5	11	28.2	9	23.1	39	36.1
	More than or equal to 60 Lux	16	23.2	16	23.2	19	27.5	18	26.1	69	63.9
Home Ventilation Size	Less than 10% of the floor area	13	26.0	11	22.0	12	24.0	14	28.0	50	46.3
	More than 10% of the floor area	14	24.1	13	22.4	18	31.0	13	22.4	58	53.7

**Table 2:** Environmental factors that pose a risk for pulmonary TB transmission.

Environmental Variables		Case		Control		Amount	*p*-Value	Odds Ratio
		n	%	n	%			
Home Ventilation Size	Less than 10% of the floor area	23	63.89	21	29.17	44	0.006	8.322
	More than 10% of the floor area	13	36.11	51	70.83	64		
Stigma Competency	High	23	74.19	37	51.39	60	0.024	9.074
	Low	8	25.81	35	48.61	43		
Lighting	Less than 60 Lux	18	50.00	21	29.20	39	0.014	9.013
	More than or equal to 60 Lux	18	50.00	51	70.80	69		

**Table 3:** Results of RAPFISH analysis of community triggering as an environmentally based pulmonary TB transmission risk control strategy.

Dimensions	Stress	R^2^	MDS Values	Sustainability Status	Monte Carlo Test	Deviant (MDS-Monte Carlo)
Ecology	0.144	0.948	72.59	Fair	71.16	1.44
Economy	0.156	0.942	63.32	Fair	62.90	0.42
Social	0.160	0.939	63.01	Fair	62.33	0.68
Institution	0.153	0.944	61.59	Fair	61.27	0.33
Technology	0.134	0.953	84.53	High	82.08	2.45
	Average		69.01	Fair		

## Data Availability

The datasets generated for this study can be requested from the corresponding author upon reasonable request.

## Supplementary Materials

The following supporting information can be downloaded at https://www.mdpi.com/article/10.3390/ijerph23081063/s1, Figure S1: LEVERAGE Analysis of Community-Triggering; Figure S2: Sustainability Status of Community-Triggering.

## Appendix A. TB Stigma (Number of Correct Answers/Number of Items × 100)

**Question**	**Correct Answer (Four Options)**
TB stigma is defined as ….	Form of refusal or discrimination to people with TB
One of the factors that is affected to TB stigma is ….	Limited education through the community
TB stigma may impact to ….	TB patient that is fear to seek medication which it could transmit to the other
Best way to prevent TB stigma is ….	To socialize correct information about TB
The role of community in TB control includes ….	Encourage people with TB and maintain healthy environments
Why is it important to eliminate TB stigma?	So the community could help without hesitate

## Appendix B. Expert Assessment Instrument for the Sustainability of Community-Triggering Programs

**Question**	**Best Answer (Three Points)**
Dimension: ECOLOGY	
Ecological conditions such as house layout, vegetation, and building density affect ventilation and natural lighting [35,36]	Very influential
Community triggering promotes changes in the physical environment of the house that are friendly to lung health (ventilation, lighting) [35,36]	Very significant
Behavioral changes from community triggering (e.g., opening windows) are ecologically sustainable in the long term [35,36]	Highly sustainable
The program is tailored to local ecological conditions (climate, wind direction, land conditions) [37]	Highly sustainable
Triggering design encourages environmental conservation (tree planting, environmentally friendly local materials) [36]	Highly sustainable
The program has the potential to improve the long-term quality of residential ecosystems [36]	Very potential
Community involvement strategies strengthen local ecological awareness in maintaining healthy homes [38]	Very strengthening
	
Dimension: ECONOMIC	
The cost of home improvements after the trigger (ventilation, lighting) is affordable for low-income communities [35,36]	Very affordable
The program provides financial support/economic incentives for home improvements [39]	Very good/Fully supportive
Ventilation and lighting changes provide long-term economic benefits (energy savings, health) [35,36]	Very good/Fully supportive
The program stimulates local economic activity (craftsmen, local materials, health MSMEs) [37]	Very good/Fully supportive
Program sustainability without dependence on external funding [39]	Very sustainable
Community triggering is economically feasible for replication in resource-limited areas [37]	Very feasible
Training/socialization costs are efficient compared to their economic impact [37]	Very feasible
	
Dimension: SOCIAL	
The program increases community awareness of the importance of ventilation and lighting in preventing the risk of pulmonary TB transmission [35,36]	Very good
The program fosters active community participation in maintaining healthy homes [38]	Very good
Community motivators strengthen social cohesion (cooperation, communication between residents) [40]	Very good/Fully supportive
Community perception of behavioral changes (e.g., opening windows every day) is positive [38]	Very good/Fully supportive
This program reduces the stigma against people with pulmonary TB [36]	Very good/Fully supportive
This program can be sustained through local leadership/community leaders	Very significant
	
Dimension: INSTITUTIONAL	
This program can be integrated into community social activities (PKK, Posyandu, social gathering)	Very possible
This program can be integrated into local institutional systems (subdistrict, community health center, PKK, RT/RW)	Very good
Local institutions have a long-term commitment to supporting ventilation and lighting triggering [35,36]	Very good/Fully supportive
Regulations/SOPs/guidelines are available for the sustainable implementation of triggering [36]	Widely implemented
Institutional capacity (human resources, structure, coordination) is sufficient to sustain the program	Very good/Fully supportive
Monitoring and evaluation mechanisms exist from the institution regarding program sustainability [35]	Exist and running effectively
The institution is capable of establishing cross-sector partnerships (services, NGOs, universities) [35]	Very good/Fully supportive
Involvement of local facilitators (cadres, community leaders) supported by local institutions [38]	Highly facilitated and empowered
	
Dimension: TECHNOLOGY	
Technology (cell phones, digital applications, online reporting) has been used in TB community-triggering activities [19,41]	Very good/Fully supportive
Technology assists in the implementation of triggering activities in coastal and highland areas [19,29]	Very good/Fully supportive
Internet networks and electricity access adequately support the use of technology at program locations [41,42]	Very good/Fully supportive
Cadres, officers, and community leaders can use basic technology (WhatsApp, digital photos, online forms)	Very good/Fully supportive
Technology use has the potential to continue independently by the community after the program is completed [43]	Very good/Fully supportive
Activity reporting systems (applications, WhatsApp, Google Forms, etc.) run routinely and are accessible [41]	Very good/Fully supportive
Data from technology is used for TB control evaluation and planning [36,43]	Very good/Fully supportive
Village governments, community health centers, or agencies support the use of technology, especially in hard-to-reach areas	Very good/Fully supportive
Long-term resources such as funds, internet quota, phone credit, or supporting devices are available for cadres/officers [42]	Very good/Fully supportive
Technology helps improve public understanding and encourages behavior change related to TB [15,41]	Very good/Fully supportive
