# Supplementary material for: Risk Factors, Distribution Patterns, and Their Implications for Environmentally Based Tuberculosis Transmission Risk Control Strategies
Source: Int J Environ Res Public Health. 2026 Aug 17;23(8):1063. doi: 10.3390/ijerph23081063 (PMC13512747; doi:10.3390/ijerph23081063)
Supplement: Supplementary file 1 [file ijerph-23-01063-s001.zip › ijerph-4165179-supplementary.pdf]

1.1. LEVERAGE Analysis of Community-Triggering

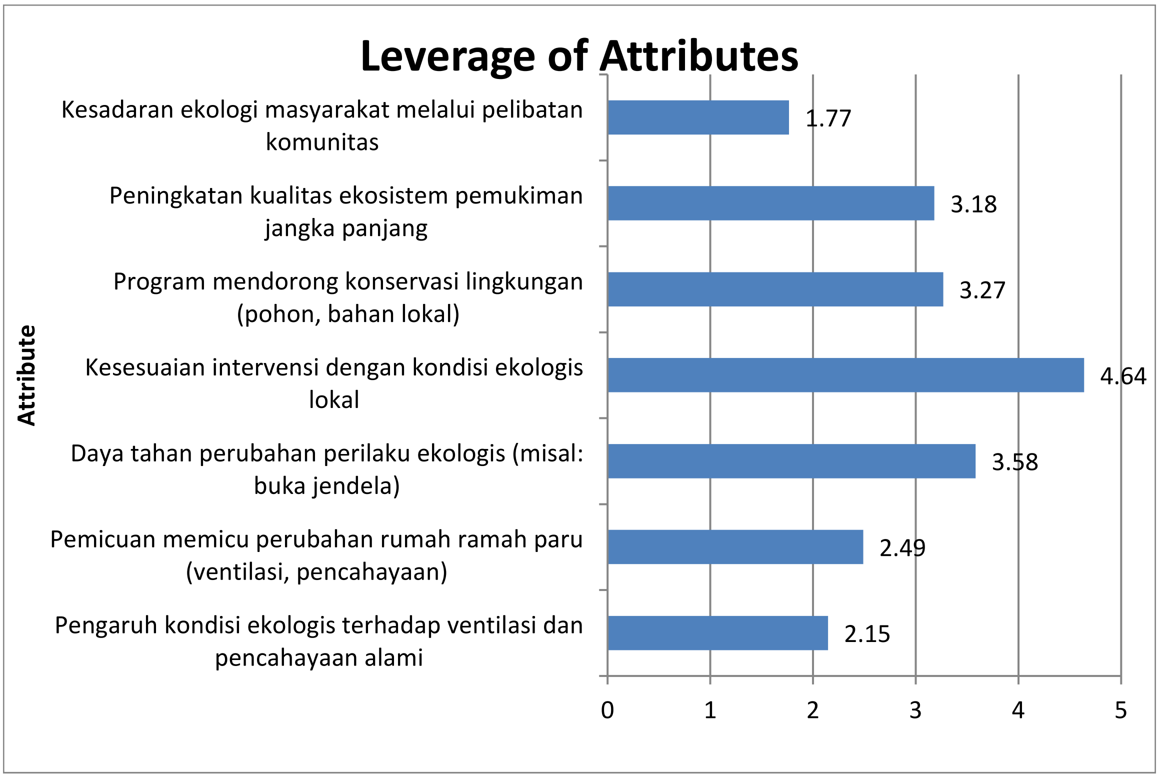

(a)

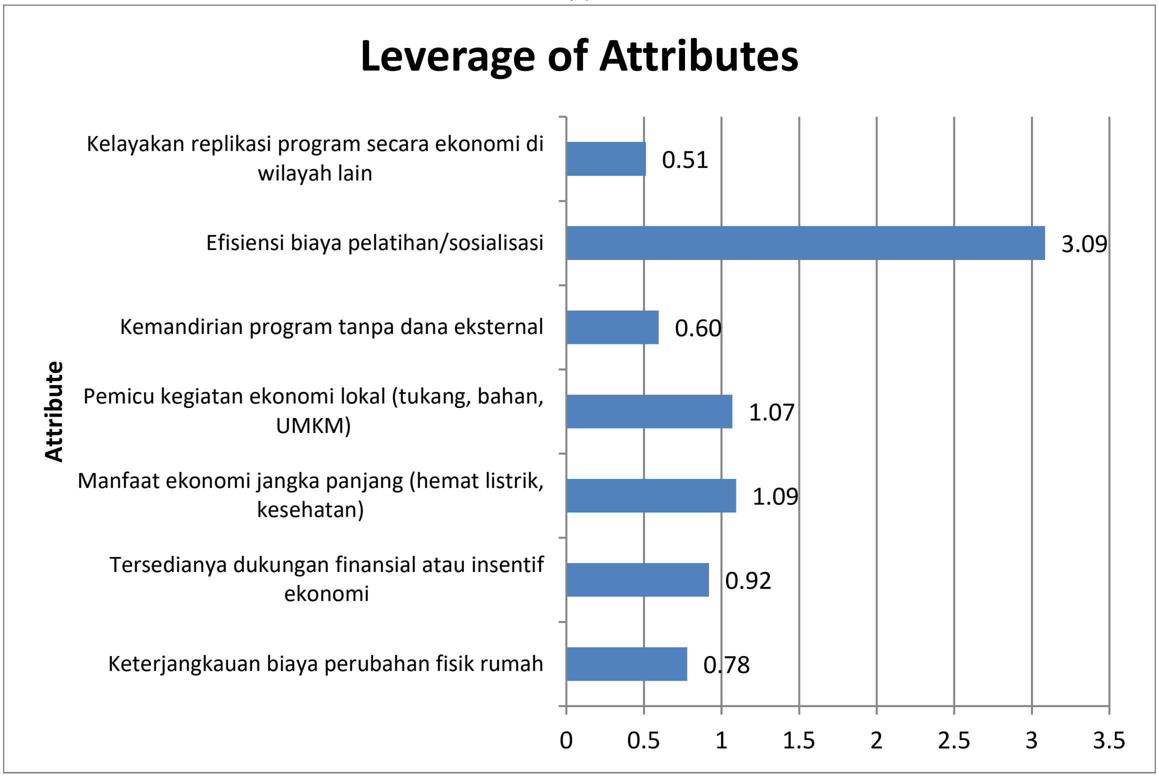

(b)

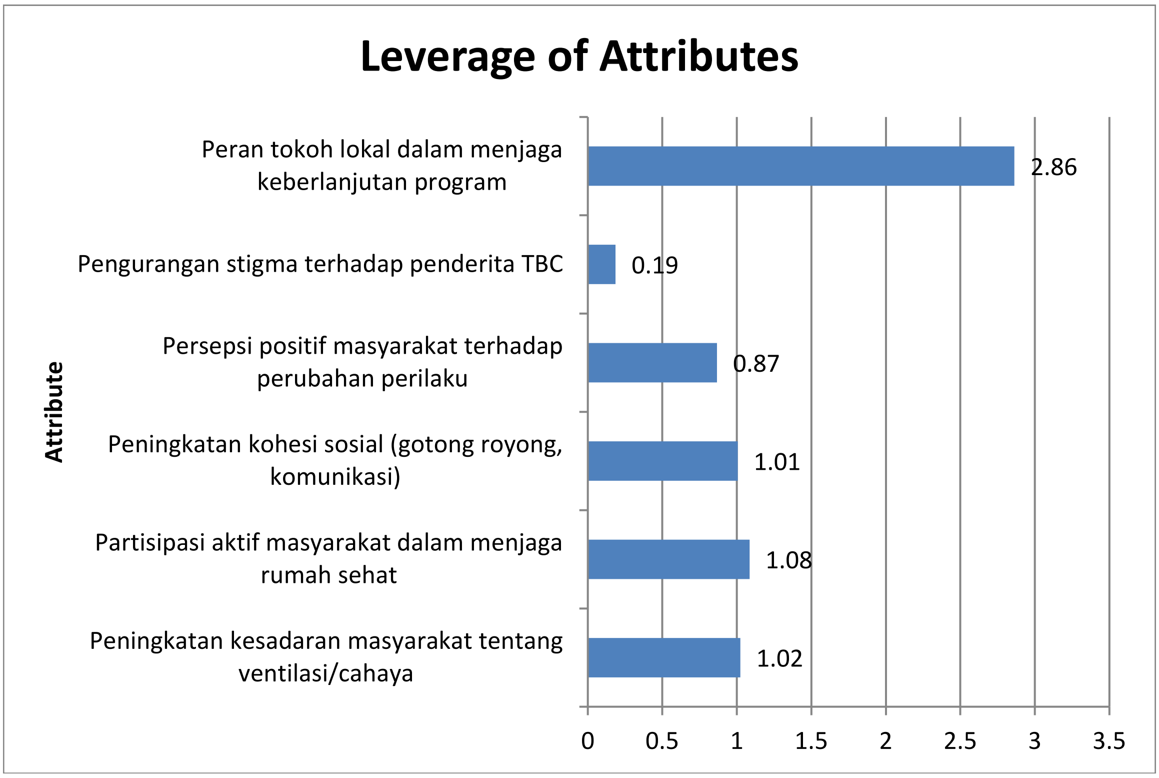

Leverage of Attributes
Peran tokoh lokal dalam menjaga keberlanjutan program
Pengurangan stigma terhadap penderita TBC
Persepsi positif masyarakat terhadap perubahan perilaku
Peningkatan kohesi sosial (gotong royong, komunikasi)
Partisipasi aktif masyarakat dalam menjaga rumah sehat
Peningkatan kesadaran masyarakat tentang ventilasi/cahaya
Attribute
2.86
0.19
0.87
1.01
1.08
1.02
0
0.5
1
1.5
2
2.5
3
3.5

(c)

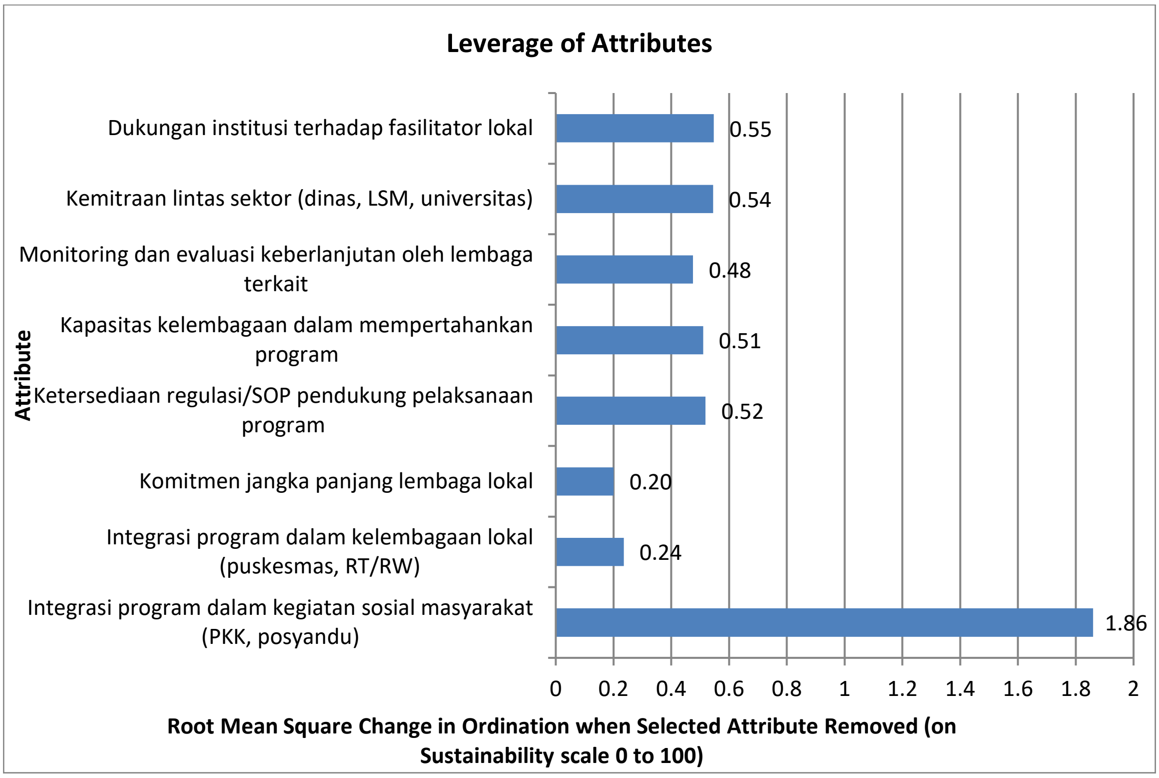

Leverage of Attributes
Dukungan institusi terhadap fasilitator lokal
Kemitraan lintas sektor (dinas, LSM, universitas)
Monitoring dan evaluasi keberlanjutan oleh lembaga terkait
Kapasitas kelembagaan dalam mempertahankan program
Ketersediaan regulasi/SOP pendukung pelaksanaan program
Komitmen jangka panjang lembaga lokal
Integrasi program dalam kelembagaan lokal (puskesmas, RT/RW)
Integrasi program dalam kegiatan sosial masyarakat (PKK, posyandu)
Attribute
0.55
0.54
0.48
0.51
0.52
0.20
0.24
1.86
0
0.2
0.4
0.6
0.8
1
1.2
1.4
1.6
1.8
2
Root Mean Square Change in Ordination when Selected Attribute Removed (on Sustainability scale 0 to 100)

(d)

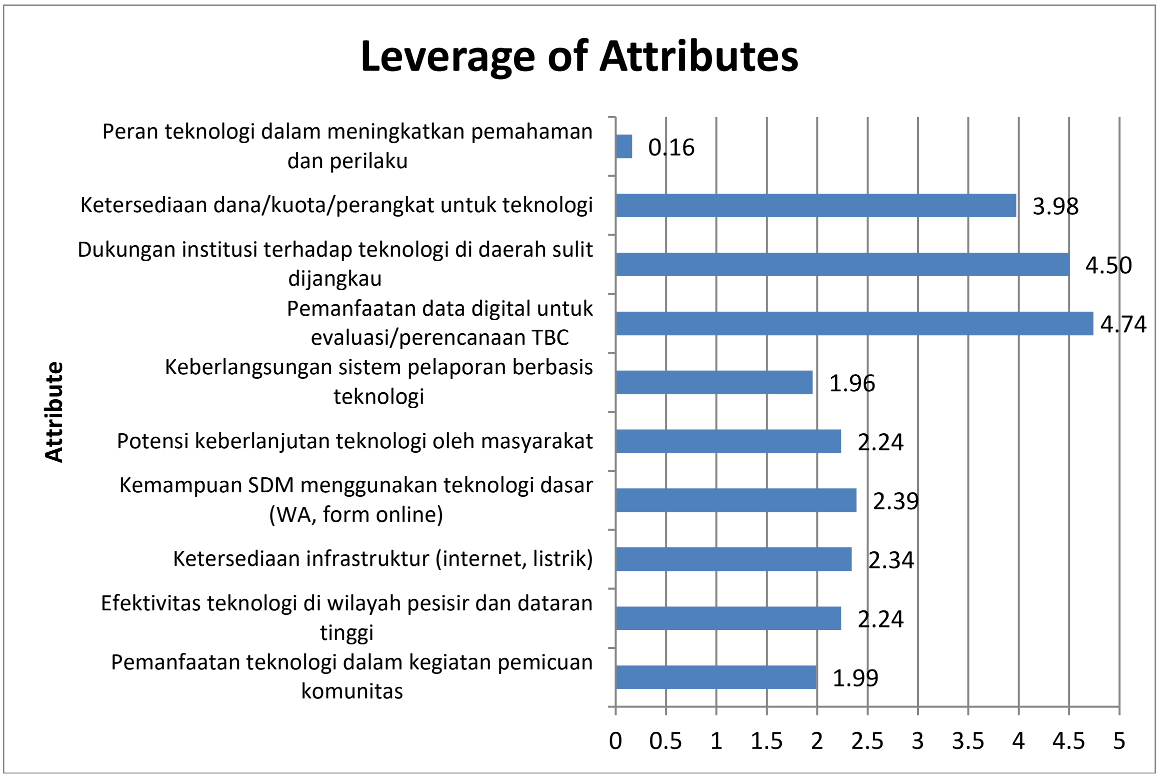

(e)

Figure S1. Results of the LEVERAGE Analysis of Community-triggering from: (**a**) Ecological; (**b**) Economic; (**c**) Social; (**d**) Institutional; and (**e**) Technological Dimensions.

The results of the leverage analysis on the ecological dimension identified two attributes with the most significant influence on the level of sustainability. These attributes are "Suitability of the intervention to local ecological conditions," which had the highest leverage value of 4.64, followed by "Durability of ecological behavior change (e.g., opening windows)" with a value of 3.58 (Figure S1a). Based on the LEVERAGE analysis of the Economic dimension, the main attribute with the highest leverage is "Cost efficiency of training/socialization" with a score of 3.09. Furthermore, the attribute "Long-term economic benefits (energy savings, health)" has a score of 1.09 (Figure S1b). The results of the leverage analysis on the social dimension show that "The role of local figures in maintaining program sustainability" is the attribute with the highest leverage, 2.86. Other factors, such as "Active community participation" (1.08) and "Increased social cohesion" (1.01), also have an influence, but at a much lower level (Figure S1c). The results of the LEVERAGE analysis on the Institutional dimension indicate that the attribute with the most significant leverage is "Program integration in community social activities (PKK, posyandu)," with a value of 1.86. The influence of this attribute is significantly more dominant than that of other institutional factors, such as "Institutional support for local facilitators" (0.55) and "Cross-sector partnerships" (0.54) (Figure S1d). Based on the leverage analysis on the Technology dimension, three attributes were identified as having the highest leverage. These attributes are "Utilization of digital data for TB evaluation/planning" (4.74), "Institutional support for technology in hard-to-reach areas" (4.50), and "Availability of funds/quota/equipment for technology" (3.98). These factors are much more influential than other attributes, such as "Ability of human resources to use basic technology" (2.39) (Figure S1e).

1.2. Sustainability Status of Community Triggers

Figure S2 illustrates the sustainability status position of community triggers across each dimension, which is confirmed by these Monte Carlo sensitivity tests.

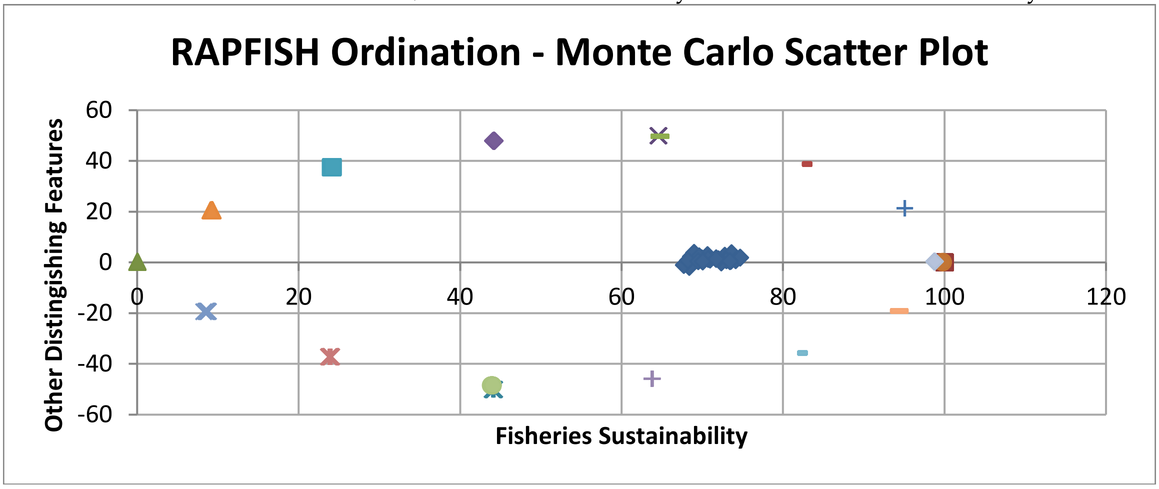

(a)

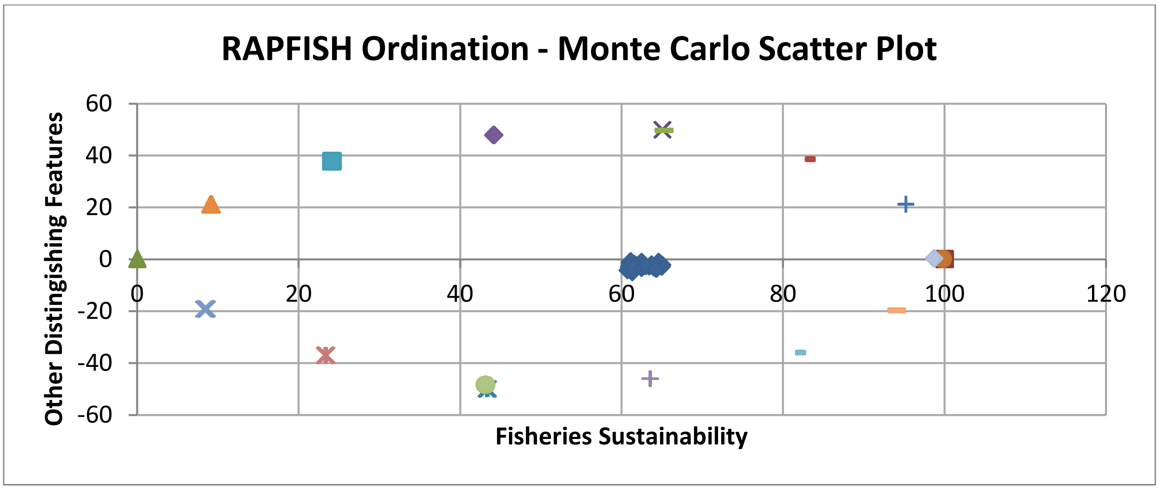

(b)

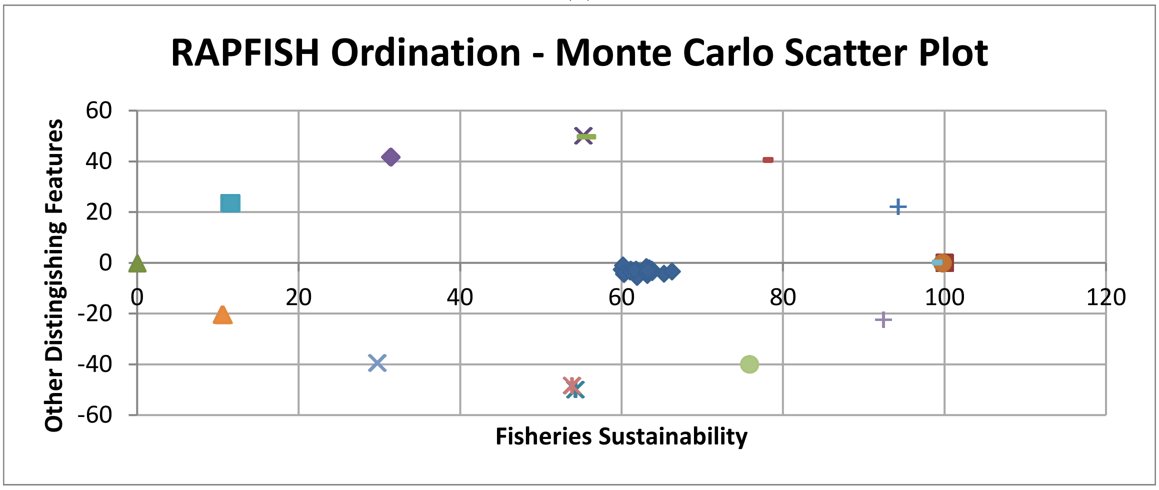

(c)

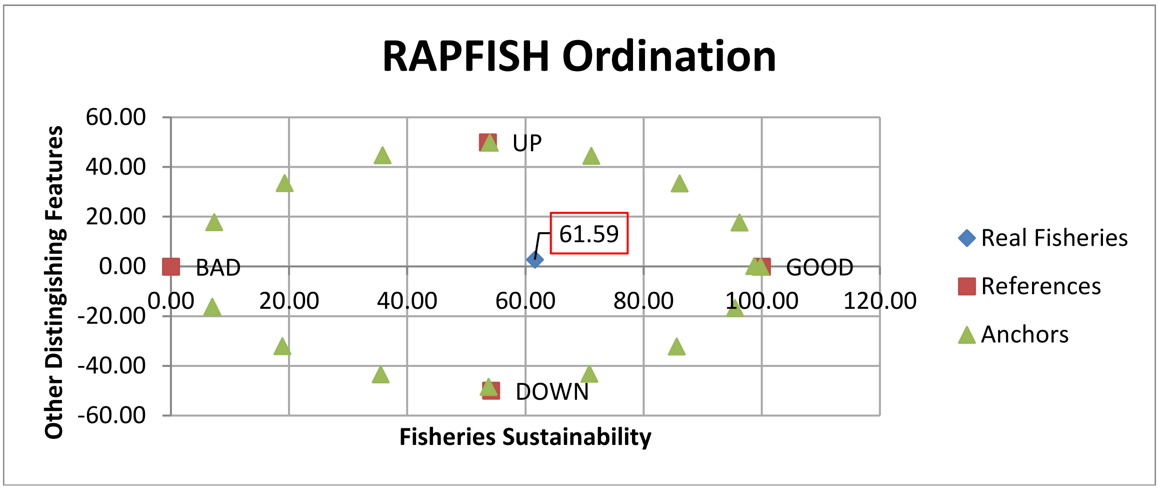

(d)

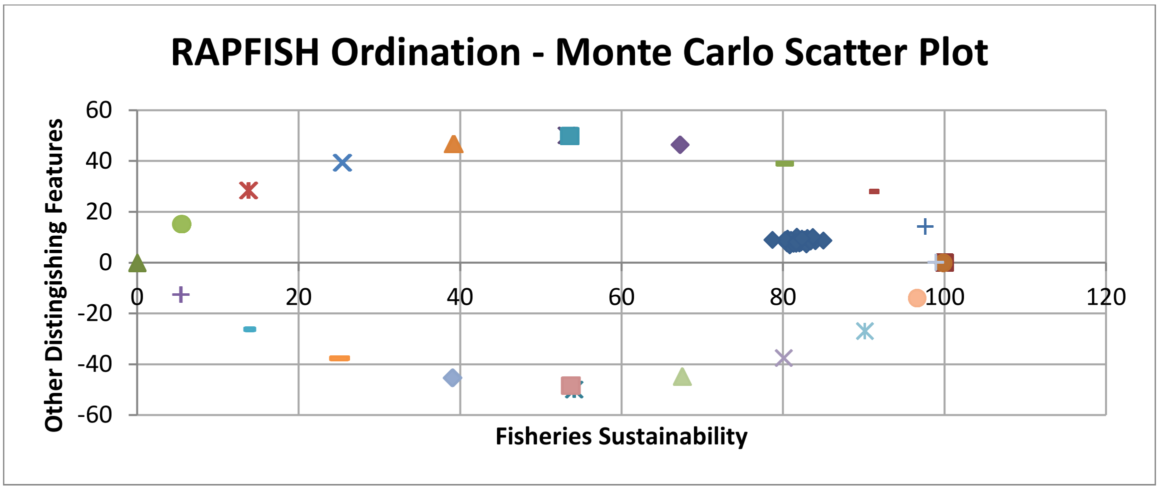

(e)

Figure S2. Sustainability status position of community triggers from: (**a**) ecological; (**b**) economic; (**c**) social; (**d**) institutional; and (**e**) technological dimensions, based on Monte Carlo analysis.
